# Artificial selection reveals the energetic expense of producing larger eggs

**DOI:** 10.1186/s12983-016-0172-y

**Published:** 2016-08-23

**Authors:** Joel L. Pick, Pascale Hutter, Christina Ebneter, Ann-Kathrin Ziegler, Marta Giordano, Barbara Tschirren

**Affiliations:** Department of Evolutionary Biology and Environmental Studies, University of Zurich, Winterthurerstrasse 190, 8057 Zurich, Switzerland

**Keywords:** Life history evolution, Maintenance of variation, Cost of reproduction, Egg size, Maternal investment, Oxidative stress

## Abstract

**Background:**

The amount of resources provided by the mother before birth has important and long-lasting effects on offspring fitness. Despite this, there is a large amount of variation in maternal investment seen in natural populations. Life-history theory predicts that this variation is maintained through a trade-off between the benefits of high maternal investment for the offspring and the costs of high investment for the mother. However, the proximate mechanisms underlying these costs of reproduction are not well understood. Here we used artificial selection for high and low maternal egg investment in a precocial bird, the Japanese quail (*Coturnix japonica*) to quantify costs of maternal reproductive investment.

**Results:**

We show that females from the high maternal investment lines had significantly larger reproductive organs, which explained their overall larger body mass, and resulted in a higher resting metabolic rate (RMR). Contrary to our expectations, this increase in metabolic activity did not lead to a higher level of oxidative damage.

**Conclusions:**

This study is the first to provide experimental evidence for metabolic costs of increased per offspring investment.

## Background

The environment experienced during early development can have significant and long-lasting consequences for an individual’s phenotype [1, 2]. Mothers are in a unique position to influence these early life conditions through, for example, the quantity and quality of resources they provide to their offspring [3]. Despite the positive effects of increased maternal investment on offspring fitness [3, 4], there is a large amount of variation in reproductive investment seen in natural populations [5, 6]. Life-history theory predicts that this variation is maintained by trade-offs between the benefits of increased investment for the offspring and the associated costs to the mother [7–9].

However, despite being a central tenant of life history theory, the mechanisms underlying these costs of reproduction are not well understood [10, 11]. Various mechanisms have been proposed to mediate the costs of reproduction. Costs may, for example, occur because females reallocate energy or resources from self-maintenance to reproduction [12]. If these reallocations cannot fully cover the increased energetic demands during reproduction, females have to increase their rate of energy conversion, through an increase in metabolic rate. This, in turn, can lead to a higher production of reactive oxygen species (ROS), produced in the mitochondria as a by-product of cellular respiration [13]. When not balanced by antioxidant defences, high levels of ROS are associated with cellular damage, referred to as oxidative stress [13], which has been proposed to be a key mediator of life-history trade-offs [14, 15]. Furthermore, an increased energetic demand may lead to extended food searching and so a higher predation risk [16, 17].

To date, most studies that explored the costs of reproduction, and in particular the costs of per offspring investment, are correlative [15] and therefore cannot reveal trade-offs [18]. In birds, and especially in precocial species that do not show extensive parental care after hatching, per offspring maternal resource investment is reflected in the size of the egg [19], which varies considerably in natural populations [20]. Although egg production per se is known to be an energetically demanding process [12, 21], few studies have explicitly quantified the costs of increased per offspring investment and those that have mainly focused on egg size-number trade-offs, for which there is little evidence [22–25].

Maternal egg investment (i.e. per offspring investment) is notoriously difficult to alter experimentally and, to our knowledge, no study has manipulated maternal egg investment and examined the costs to the mother. To address this gap, we established artificial selection lines for high and low maternal egg investment in a precocial bird, the Japanese quail (*Coturnix japonica*). Through artificial selection, we experimentally manipulated egg size, producing females that differ genetically in how much they invest in their eggs (relative to their body size). This selection resulted in a correlated response in resource investment (dry egg components), but there was no evidence for a trade-off with the number of eggs laid [24]. Here we show that the mothers’ reproductive organ size increased in line with the level of their reproductive investment, but there was no evidence for a reallocation of lipid or protein reserves. The increase in reproductive organ size in high investment mothers was associated with an increase in metabolic rate, but no apparent increase in oxidative damage. Our study suggests that metabolic costs for the mother may play an important role in the maintenance of variation in reproductive investment observed in natural populations.

## Methods

### Study population and selection lines

For this study we used established replicated, divergent Japanese quail selection lines for high and low maternal egg investment (see [24] for a detailed description of the selection procedure). In brief, we selected for high and low relative egg size (i.e. egg size corrected for female body size), by incubating eggs from the highest and lowest 25 % of females from a base population (generation one), creating high investment and low investment lines respectively. In subsequent generations we selected the most extreme 50 % of females within each line. This procedure was repeated twice to create two independent replicates per line (i.e. High 1 / Low 1, High 2 / Low 2). As well as originating from the same base population, high and low investment line birds within a replicate were bred at the same time, meaning that they were all of the same age and experienced the same environmental conditions. By generation four, the lines differed in absolute egg size by 1.2 standard deviations (High investment: 12.46 ± 0.94 g (mean ± SD); Low investment: 11.12 ± 0.91 g; [24]). The quantity of dry components in the egg (i.e. lipids and protein) responded positively to selection on relative egg size (i.e. larger eggs contained more resources), whilst the rate of egg laying did not change between the two lines as a consequence of selection [24], suggesting that females of the high investment line do not compensate for laying larger eggs by laying fewer or lower quality eggs. Furthermore, this increase in resource investment had a positive effect on the size and early survival of offspring [26].

The birds were kept at the University of Zurich in outdoor aviaries (5 × 7.5 m each). For data collection, females were brought into cages (122 × 50 × 50 cm) within our breeding facility (see below for details about different groups). The bottom of the cages was filled with sawdust, and contained a house, a raised sandbath and ad libitum food, water, grit and shell. Reproduction in quail is strongly influenced by photoperiod [27], so we can manipulate the breeding status of a female by controlling the daylength within our breeding facility. Breeding (i.e. egg-laying) was induced by keeping females on a 16:8 h light:dark cycle, whilst non-breeding birds were kept on a 10:14 h light:dark cycle. At all times our breeding facility was maintained at approximately 20 °C. When entering the cages, body mass (to nearest 1 g) and tarsus length (to nearest 0.1 mm) were measured. Eggs were collected each morning and weighed to the nearest 0.01 g (hereafter referred to as egg size).

### Body composition

We dissected breeding females (aged between 38 and 43 weeks) from the fourth generation of the selection lines to investigate differences in body composition between the high (N: High 1 = 15; High 2 = 20) and low (N: Low 1 = 16; Low 2 = 14) investment lines. Females were kept in cages for 4 weeks with a male, and then kept for three to six days in female pairs before dissection (as part of a separate experiment).

The day before dissection, all cages were checked every hour up until one hour before the lights were switched off (21:00) and for every female it was recorded when the egg was laid. The following day females were euthanised, where possible 18 h after laying to standardise the stage of egg production. Body mass was measured before euthanisation. Oviduct, ovary (including yolky follicles), oviductal egg, liver and pectoral muscles (pectoralis and supracoracoideus) were dissected out and weighed (wet mass to nearest 0.01 g). Preliminary data showed that wet and dry masses are highly correlated (oviduct: *r* = 0.927, N = 32, *P* < 0.001; liver: *r* = 0.890, N = 32, *P* < 0.001; pectoral muscle: *r* = 0.977, N = 14, *P* < 0.001). In the second replicate we also weighed the fat in the body cavity (omentum, N = 34 females).

The liver is the site of yolk precursor synthesis [28] and so is expected to change proportionally to egg size. The pectoral muscles and body fat were dissected to test for a potential reallocation of resources from organs involved in flight ability [29] and lipid storage, respectively, to reproduction. Although Japanese quail feed and nest on the ground, flight is a vital function in this species, both for their escape response and long-distance migration [30].

To obtain a baseline from which to interpret the differences in organ sizes of breeding individuals between the selection lines (see above), we dissected ten non-breeding females from the unselected base population (aged between 24 and 26 weeks; same founders as selection lines) as described above and compared them to the breeding females from the selection lines. Given the limited number of females from the selection lines, it was not possible to use non-breeding females from the selection lines for this comparison. We expected the differences between high investment and low investment females to mirror (although to a lower magnitude) those between breeding and non-breeding females.

### Metabolic rate

We measured the metabolic rate of females from the fifth generation of the high investment (N: High 1 = 7; High 2 = 7) and low investment (N: Low 1 = 7; Low 2 = 8) lines. These females were measured twice, once in breeding condition (aged between 14 and 33 weeks) and once, 11 weeks later, in non-breeding condition. Metabolic rate measurements began 5 days after females were put into cages (at which point they were already in breeding or non-breeding condition). These measurements took place over five nights, with six females being measured each night (ensuring that the lines were balanced over the nights). Food was withdrawn from the cages for two to three hours before the measurements started to ensure a post-absorptive state. Females were weighed before being put into respirometry chambers (3.9 l plastic containers; 234 × 165 × 165 mm; Lock and Lock, Hanacobi Co. Ltd., Korea) and weighed again in the morning. The chambers were covered by dark material and lights in the windowless room were switched off. The temperature was kept at 24 - 27 °C, which is within the thermo-neutral zone for this species [31]. We measured the rate of oxygen consumption (VO_2_) using a flow-through respirometry system (Sable Systems International, Las Vegas, USA). Our setup consisted of eight metabolic chambers, six containing quail and two as controls. Air was pumped from the room into each chamber by an eight-channel mass flow meter system (Flowbar-8 Mass Flow Meter/Pump FB-8–1, Sable Systems International, Las Vegas, USA). Air was sampled from one chamber at a time (Multiplexer Intelligent RM-8–2, Sable Systems International, Las Vegas, USA), dried (magnesium perchlorate, Sigma-Aldrich, USA) and analysed (Foxbox, Sable Systems.

International, Las Vegas, USA). The mean flow rate across the nights was 1671 ± 16 mL min^−1^. We recorded O_2_, CO_2_, flow rate and temperature in consecutive 45 min periods throughout the course of the night. During these 45 min periods, all eight chambers were measured once for five minutes. One control box was measured twice, once at the beginning and once end of each period, and the other control box was measured once in the middle of each period. As the equipment took a certain time to adjust between chambers, we excluded the first 100 s of each reading, leaving 200 s per reading (with 14–18 readings per bird). We regressed all control chamber readings for both CO_2_ and O_2_ against time within a 45 min period, and used this to predict baseline gas levels for chambers containing quail during the same 45 min period.

These baseline readings were then used to calculate VO_2_:

$$ V{O}_2=FR\frac{\left(Fi{O}_2-Fe{O}_2\right)-Fe{O}_2\left(FeC{O}_2-FiC{O}_2\right)}{1-Fe{O}_2} $$

[32], where FiO_2_ and FiCO_2_ are the baseline O_2_ and CO_2_ readings (divided by 100), respectively, FeO_2_ and FeCO_2_ are O_2_ and CO_2_ readings (divided by 100), respectively, for the chamber in question and FR is the flow rate. We define metabolic rate as the lowest, stable VO_2_ reading of a resting animal, in a post-absorptive state, within its thermalneutral zone. Typically this is described as the basal metabolic rate (BMR), but given that half of our measurements were of females in breeding condition, and so physiologically ‘active’, we use the broader term resting metabolic rate (RMR; following [33]). RMR therefore represents the basic cost of living. To obtain RMR we calculated the mean VO_2_ of the lowest, stable one minute during the whole night for each bird.

This measurement of RMR was highly correlated with mean VO_2_ across the whole night (*r* = 0.977, N = 64, *P* < 0.001). Repeatability of RMR, based on six birds that were measured twice in non-breeding condition (with 5 days between measurements), was high after correcting for the overall difference between nights (*r* = 0.868 ± 0.106, *F*_5,6_ = 14.14, *P* = 0.003).

### Oxidative damage

Three days after the metabolic rate measurements we took a blood sample from all females from the brachial vein using heparinised capillary tubes. Samples were kept on ice until centrifugation (5 min at 20 °C and 2000 × g). Plasma was then separated and frozen at −80 °C until analysis. As a measure of oxidative damage we quantified the plasma concentration of reactive oxygen metabolites (ROMs) using the dROMs test (Diacron International, Grosseto, Italy). This is a colorimetric assay, which measures intermediate oxidative damage molecules (mainly hydroperoxides; [34]) that are produced by the peroxidation of a diverse range of biomolecules [35]. Our analysis followed previously published protocols [36, 37]. In short 8 *μ*l plasma was diluted with 200 *μ*l of a solution containing acetate buffer (pH 4.8) and an aromatic alkyl-amine (chromogen). The samples were incubated at 37 °C for 75 min, centrifuged and the supernatant was pipetted onto a microplate. The absorbance was then read with a spectrometer (Multiskan Spectrum, ThermoFisher, Vantaa, Finland) at a wavelength of 505 nm. All samples were run in duplicate. Results were calculated as mM of *H*_2_*O*_2_ equivalents. There was a high repeatability of ROMs measures within samples (*r* = 0.993 ± 0.002, *F*_55,56_ = 282.61, *P* < 0.001). The inter-assay coefficient of variation was 8.12 %, and the intra-assay coefficient of variation was 1.55 %. In order to correct for plate differences in ROMs, we centered all samples from a plate on the control samples for that plate. One low line female had blood taken only once, and so was excluded from the oxidative damage analyses.

### Statistical analysis

We compared differences in total body mass (at time of dissection), reproductive organ mass, non-reproductive mass, liver, fat and pectoral muscle mass, as well as metabolic rate and oxidative damage between the selection lines and between non-breeding and breeding individuals. Reproductive organ mass included oviduct mass, ovary mass and the mass of yolky follicles. Non-reproductive mass was calculated as the total body mass minus reproductive organ mass and oviductal egg mass. All measures were log transformed prior to analysis to account for scaling effects on variance.

To test for differences in body composition between non-breeding and breeding females, we used two sample t-tests and a Welch/Satterthwaite approximation for the degrees of freedom due to unequal sample sizes and variances. One non-breeding female was excluded from the analysis, as during dissection it was clear from the state of her ovary and oviduct that she had started to come into breeding condition.

To test for differences in body composition between breeding females from the high and low investment lines, we used linear models, including selection line and replicate as factors. Tarsus length (cubed prior to log transformation) was included as a covariate to account for body size differences among females. For the analysis of total body mass and reproductive organ mass, we included only the 55 females that were dissected approximately 18 h after laying, as the mass of the reproductive organs varies with the stage of egg development (N: High 1 = 12; High 2 = 19; Low 1 = 13; Low 2 = 11).

Body mass (i.e. mass when entering the metabolic chamber), metabolic rate and oxidative damage of the females measured in generation five, were measured twice, once in breeding and once in non-breeding condition. To test whether the change in these traits between non-breeding and breeding condition was different between the lines, we ran linear mixed models with selection line, breeding status and replicate as factors as well as the interaction between selection line and breeding status. Age and tarsus length were included as covariates and again tarsus length was cubed prior to log transformation. Female ID was included as a random effect. In the metabolic rate models, we also included measurement date as a random effect to account for stochastic differences in RMR measurements between nights.

If the *level* of reproductive investment affects the increase in body mass, metabolic rate and/or oxidative damage, we predict to see a significant interaction effect between line and breeding status in all of these models. If the increase in metabolic rate is driven by an increase in body mass, then we predict to find this interaction when correcting for body size (tarsus length) but not when correcting for body mass. Similarly, if the increase in oxidative damage is driven by an increase in metabolic rate, we predict to no longer find an interaction effect between breeding status and line when correcting for metabolic rate or body mass. To test these hypotheses we ran an additional model for metabolic rate with log transformed body mass as a covariate instead of tarsus length and two additional models for oxidative damage including log transformed body mass and log transformed metabolic rate, respectively. In these models the added covariate was always retained in the model.

Additionally we used paired t-tests to test whether body mass, metabolic rate and oxidative damage differed between individuals in breeding and non-breeding condition.

We included these tests to demonstrate the magnitude and direction of the difference between breeding and non-breeding birds, and to allow a comparison with the body composition data. Finally we tested whether the within individual change between non-breeding and breeding condition in all three measures correlated with each other, as well as with mean egg size and tarsus length.

All analyses were run in R (3.0.3, [38]). In all models, we performed backward stepwise deletion of non-significant terms. Significance was determined using F statistics in linear models and likelihood ratio tests with one degree of freedom in mixed effects models. We present means ± SD.

## Results

### Body composition

Breeding females (from both selection lines) were significantly heavier than non-breeding females (non-selected birds; Table 1). This mass difference between breeding and non-breeding females (30 g) was mainly due to an increase in reproductive organ mass (15.18 ± 1.73) plus oviductal egg mass (11.72 ± 1.14; total = 26.36 ± 3.88 g).

**Table 1:** Body composition, metabolic rate (RMR) and oxidative damage (ROMs) of breeding and non-breeding females

Trait	Breeding	Non Breeding	*t*	df	*P*
Generation 4					
Body Mass					
Total Body Mass (g)	288 ± 35	258 ± 28	**2.84**	**10.90**	**0.016**
Non-Repro. Mass (g)	262 ± 33	258 ± 28	0.36	11.07	0.724
Body Size					
Tarsus Length (mm)	40.0 ± 1.4	39.8 ± 1.1	0.38	12.09	0.712
Reproductive and Associated Organs					
Repro. Organs (g)	15.18 ± 1.73	0.31 ± 0.12	**39.30**	**8.36**	<**0.001**
Liver (g)	7.98 ± 1.06	4.66 ± 0.80	**9.78**	**9.60**	<**0.001**
Protein and Lipid Reserves					
Pectoral Muscles (g)	51.85 ± 7.53	51.38 ± 4.67	0.08	14.43	0.939
Body Fat (g)	4.96 ± 3.00	8.59 ± 3.94	−**3.29**	**21.45**	**0.003**
Generation 5					
Body Mass (g)^1^	254 ± 20	240 ± 17	**4.20**	**28**	<**0.001**
RMR (mL O_2_ min^−1^)	6.01 ± 0.67	3.52 ± 0.37	**22.73**	**28**	<**0.001**
ROMs (mM H_2_O_2_)	0.882 ± 0.263	0.801 ± 0.228	1.22	27	0.232

Means ± SD are shown. In generation 4, females were measured once, either in breeding (*N* = 65) or in non-breeding (*N* = 10) condition. In generation 5, females (*N* =29) were measured twice, once in breeding and once in non-breeding condition. Repro. is abbreviation for Reproductive. Significant results are displayed in bold

^1^The difference in body mass between the two states is less than in generation 4 due to measuring the birds at different times of day (here the majority of females had already laid an egg)

Non-reproductive mass did not differ between non-breeding and breeding females (Table 1). Breeding females also had heavier livers and less body fat than non-breeding females, but there was no difference in pectoral muscle mass (Table 1).

**Fig. 1:**
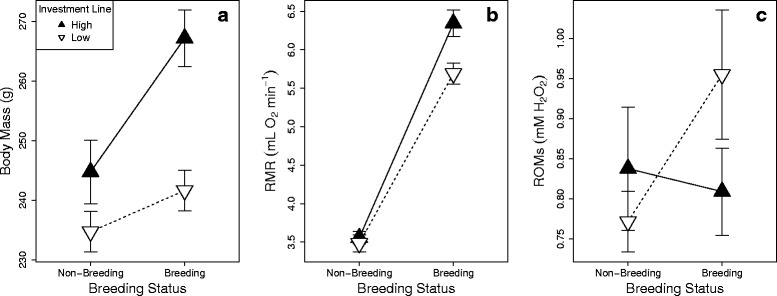
The effect of selection line and breeding status on **a**) body mass, **b**) metabolic rate (RMR) and **c**) oxidative damage (ROMs)

Similarly, high investment females tended to be heavier than low investment females when correcting for body size (generation 4; Table 2). Furthermore, the change in body mass between breeding and non-breeding condition was significantly larger in high investment females (22 ± 14 g) than low investment females (7 ± 19 g; generation 5; Table 3, Fig. 1a).

**Table 2:** Difference in body composition of breeding females between selection lines, correcting for replicate and body size

Trait	Line			Replicate			Tarsus Length		
	*F*	df	*P*	*F*	df	*P*	*F*	df	*P*
Body Mass									
Total Body Mass	3.33	52	0.074	0.51	51	0.480	**40.89**	**53**	< **0.001**
Non-Repro. Mass	1.92	62	0.171	0.34	61	0.561	**39.19**	**63**	< **0.001**
Reproductive and Associated Organs									
Repro. Organs	**7.15**	**51**	**0.010**	**9.32**	**51**	**0.004**	**19.73**	**51**	<**0.001**
Liver	0.94	62	0.336	0.02	61	0.891	**3.99**	**63**	**0.050**
Protein and Lipid Reserves									
Pectoral Muscles	1.65	61	0.204	**19.19**	**62**	<**0.001**	**37.09**	**62**	<**0.001**
Body Fat	0.00	32	0.958	-	-	-	0.00	31	0.994

Significant results are displayed in bold. DF is the denominator degrees of freedom. Numerator degrees of freedom was 1 in all cases. Repro. is abbreviation for Reproductive

**Table 3:** Difference between the selection lines in body mass (BM), metabolic rate (RMR) and oxidative damage (ROMs) according to breeding status

Trait	Model	Line		Status		Line x Status		Replicate		Age		Tarsus Length		Body Mass		RMR	
		*χ* ^2^	*P*	*χ* ^2^	*P*	*χ* ^2^	*P*	*χ* ^2^	*P*	*χ* ^2^	*P*	*χ* ^2^	*P*	*χ* ^2^	*P*	*χ* ^2^	*P*
BM	a	-	-	-	-	**5.29**	**0.021**	0.19	0.665	0.88	0.347	**10.73**	**0.001**	-	-	-	-
RMR	a	-	-	-	-	**4.82**	**0.028**	0.21	0.646	0.00	0.947	2.17	0.141	-	-	-	-
	b	0.16	0.688	**30.57**	<**0.001**	1.10	0.294	0.12	0.734	0.45	0.503	-	-	**30.01**	<**0.001**	-	-
ROMs	a	0.06	0.802	1.85	0.174	3.08	0.079	3.08	0.079	2.00	0.158	1.07	0.300	-	-	-	-
	b	0.28	0.596	1.87	0.172	3.20	0.074	3.44	0.064	1.69	0.193	-	-	0.02	0.880	-	-
	c	0.55	0.459	0.27	0.607	3.19	0.074	3.67	0.056	1.68	0.195	-	-	-	-	1.14	0.287

Significant results are displayed in bold. For metabolic rate and oxidative damage, several models were run, including a) tarsus length, b) body mass and c) metabolic rate as covariates. In all models df = 1

After correcting for body size, the reproductive organs were significantly heavier in high investment females than in low investment females, whereas non-reproductive mass did not differ between the lines (Table 2). Moreover, egg size was highly correlated with reproductive organ mass (*r* = 0.810, N = 54, *P* < 0.001). No differences in fat, liver or pectoral muscle mass were observed between the lines after correcting for body size (Table 2).

### Metabolic rate and oxidative damage

Females increased their resting metabolic rate by 70 % when entering breeding condition (Table 1). This change in RMR was significantly larger in high investment females (2.79 ± 0.65 mL O_2_ min^−1^) than low investment females (2.21 ± 0.54 mL O_2_ min^−1^; Table 3, Fig. 1b). When correcting for body mass instead of body size, the change in RMR did not differ between the lines, but there was still a significant difference in RMR between breeding and non-breeding individuals (Table 3). This demonstrates that the differential increase in RMR between the lines was mediated by the greater increase in body mass between non-breeding and breeding states in high investment line females.

Overall, there was no difference in oxidative damage when an individual was in breeding or non-breeding condition (Table 1). There was a trend for an interaction between line and breeding status on oxidative damage (Table 3; Fig. 1c), but in the opposite direction than predicted: the oxidative damage of low investment females tended to increase with breeding (paired *t* test: *t*_13_ = 1.96, *P* = 0.072) whilst there was no change in oxidative damage between non-breeding and breeding condition in high investment females (paired *t* test: *t*_13_ = 0.24, *P* = 0.815). When correcting for either body mass or RMR instead of body size, there was no qualitative change in the results (Table 3).

The changes in both body mass and RMR between non-breeding and breeding were highly correlated with each other, and both were correlated with egg size, but not with tarsus length (Table 4). Change in oxidative damage was not correlated with any other variable. Egg size and tarsus length were not correlated (Table 4).

**Table 4:** Correlations between individual differences in body mass, metabolic rate (RMR) and oxidative damage (ROMs) between breeding and non-breeding condition, egg size and tarsus length

	∆ Body Mass	∆ RMR	∆ ROMs	Egg Size	Tarsus Length
∆ Body Mass	-	**0.003**	0.822	**0.000**	0.309
∆ RMR	0.539	-	0.439	**0.012**	0.292
∆ ROMs	0.045	−0.152	-	0.436	0.558
Egg Size	0.726	0.483	−0.153	-	0.082
Tarsus Length	0.213	0.205	−0.116	0.318	-

Below the diagonal Pearson correlation coefficients are displayed, above the diagonal the *P* value. Significant results are displayed in bold

## Discussion

Female body mass increased when entering breeding condition. This increase was larger in females from the high investment lines than the low investment lines and was mainly driven by an increase in reproductive organ mass. Whereas an increase in body mass when entering reproductive condition has been documented in other species [39–41], this is the first experimental evidence that the magnitude of body mass change relates to the *level* of maternal reproductive investment.

Previous work has shown that in many taxa predator escape responses are negatively affected by body mass and the additional weight of carrying eggs [42–45]. Furthermore, within-female decreases in flight performance between non-breeding and breeding has been shown to be correlated with the corresponding increase in body mass [45]. As high investment females increase their body mass to a larger degree than low investment females, their predator escape response is likely more strongly compromised, given that small increases in mass have large impacts on the time taken to reach cover [46].

High investment line females also displayed a greater increase in RMR between non-breeding and breeding condition than low investment line females. This differential change in RMR was driven by body mass, but not body size, and so by the change in reproductive organ mass. Although it is generally observed that females increase both daily energy expenditure (DEE) and RMR when entering breeding condition (e.g. [47]), there is only inconsistent correlative evidence of a link between the level of maternal egg investment and DEE [48–51] or RMR [33, 47, 52]. Our study thus provides the first experimental evidence that the level of maternal egg investment leads to a proportional increase in metabolic rate. This energetic cost of increased maternal investment is likely to be severe, as egg production occurs at a time of relatively low food abundance [12, 39]. Additionally, previous studies have found that both RMR and DEE are associated with food intake and activity [12, 39, 53, 54] and that changes in RMR are compensated by changes in food intake [55]. Therefore birds with higher energetic demands will have to spend more time searching for food, which will increase their predation risk [16, 17, 56].

Surprisingly, despite an increase in RMR, we did not find a corresponding increase in oxidative damage, either between non-breeding and breeding condition or between the selection lines. If anything, there was a tendency for low investment line females to suffer a more marked increase in oxidative damage between non-breeding and breeding than high investment line females. Although oxidative stress has been proposed to be a key mediator of life-history trade-offs [14, 15], the empirical evidence for a link between reproduction and oxidative stress is equivocal [57]. Furthermore, the idea has been criticised on the basis that ROS may not be produced in direct relation to metabolic rate, with some studies even showing that high metabolic rates can lead to a proportionally lower production of ROS (reviewed in [57]). A recent meta-analysis showed that the levels of oxidative stress do not change, or if anything tend to decrease, between non-breeding and breeding individuals [58]. Furthermore, although the authors also found that oxidative damage tends to increase with reproductive effort [58], this finding is driven by mammalian studies and there is, in line with our results, no compelling evidence of this phenomenon in birds [59–61]. Moreover, high levels of oxidative stress due to increased reproductive effort should come at the cost of reduced future survival, of which there is little evidence [62]. Together, these findings raise questions about the role of oxidative stress in mediating life-history trade-offs in birds.

It is important to note, that although we use a very common measure of oxidative damage (i.e. dROMS in blood plasma [37]), our results may have been different if we measured a different biomarker or tissue [57]. Furthermore, the effects of higher RMR on oxidative status could have been masked by ad libitum food conditions, although it is not clear that food availability mediates such a link (see discussion in [57]).

The change in RMR between non-breeding and breeding was not completely explained by the increase in body mass. This additional increase in RMR might be explained by a change in body composition. Indeed, between non-breeding and breeding condition, the size of the liver, which is metabolically highly active, increased whilst body fat decreased (see also [63, 64]). The liver is the site of yolk precursor synthesis, which is the only part of egg synthesis that requires lipids [65]. Therefore, these changes in fat and liver size may reflect the general mobilisation of lipids from storage to the liver for yolk precursor synthesis and the associated biosynthetic activity [64]. However, there was no evidence that these changes differed between the selection lines. This is in agreement with previous studies that found that the amount of yolk precursors in the plasma of breeding females was not positively correlated to yolk size or composition [12, 66, 67]. Moreover, hormonally increased yolk precursor levels caused no change in egg size [68, 69] and selection on yolk precursor levels affected liver size, but not egg size or production [70]. This has lead to the suggestion that females overproduce lipid-rich yolk precursors [12]. Our results corroborate this hypothesis, showing that changes in liver size and fat storage are related to reproduction per se, rather than the level of reproductive investment (see also [63]), which may explain why lipid supplementation has little effect on egg size [64]. Thus, there is no indication for a trade-off between reproductive investment and fat reserves, or that the liver contributes to the increased metabolic rate of high investment line females. This contrasts with other taxa in which fat is a major energetic currency in the trade-off between reproduction and somatic maintenance [71].

Traditionally body mass relative to body size has been used as a measure of body condition, which is thought to represent levels of lipid reserves [72] and is usually interpreted as an environmentally determined quality trait [73]. Several studies have shown that egg size correlates with body condition (reviewed in [20]) and concluded that female nutrient reserves influence variation in egg size (e.g. [74–76]). However, our results suggest that laying larger eggs requires larger reproductive organs, which results in an increase in body mass and so the appearance of better ‘body condition’. This measure of body condition has recently been criticised [72], and our finding that females investing differently in their eggs display a difference in body mass but no difference in body fat, further shows that this measure is inappropriate in breeding females, as it may lead to the false conclusion that residual fat reserves influence reproductive output.

Pectoral muscles are often used as a source of protein during egg production [12]. Several studies have shown that females reduce the size of their pectoral muscles when they are experimentally forced to lay more eggs ([77–79]; but see [63]), in line with the idea that the availability of proteins, rather than lipids, limit egg production [64]. This reallocation of resource can have negative effects on flight ability [78], and so the ability to raise offspring [77] and evade predators (but see [80]). Despite these previous findings, we found no difference in pectoral muscle mass either between non-breeding and breeding females or between the selection lines. It is possible that there are more subtle changes in pectoral muscle composition or structure that we could not detect.

However, our measure of muscle mass is highly correlated with dry muscle mass. Moreover, all previous experimental studies that found a decrease in muscle mass with increased egg laying [77–79], muscles condition was assessed non-destructively through the use of external measurements [81, 82], which correlates strongly with the method we used [83]. Furthermore, our method has been used to demonstrate a decrease in muscle mass between non-breeding and breeding condition [84], showing that the method is sensitive enough to detect a reallocation, if one was present. One explanation for the lack of a reallocation of resources from muscle tissue to reproduction could be that, in our study, birds had access to ad libitum food and so protein may not have been limiting. However, previous studies have found this reallocation under similar conditions [78, 84, 85], showing that trade-offs between reproduction and muscle maintenance can also be detected in captivity. We did not measure leg muscle mass, which is likely important for locomotory function in ground living birds such as quail.

However, it seems unlikely that a protein reallocation would be confined to the leg muscle, especially given that the pectoral muscle is much larger. Overall, our results therefore suggest that protein reallocation is not an obligate response to reproduction or the level of reproductive investment.

Several authors have suggested that the cost of increased reproductive investment may be passed to the offspring, rather than being dealt with by the mother [62, 86]. For example, a recent study showed that food supplemented females experience lower oxidative damage, but do not provision their eggs with more antioxidants [87], suggesting a prioritisation of self-maintenance. Similar results have been found in other taxa (e.g. [61, 88, 89]), and may explain why, across studies, females do not appear to suffer survival costs as a consequence of experimentally increased reproductive investment [62]. Furthermore, females may experience a trade-off between reproduction and functions that we have not measured in our study, such as immune function [90] or brain size [91]. Testing for such additional costs, as well as for differences in egg constituents [87] and the oxidative stress of offspring [92] between the lines will thus give greater resolution to our understanding of the costs of per offspring investment.

## Conclusions

Our study provides experimental evidence that increased female egg investment is associated with an increase in reproductive organ mass, leading to an increase in both body mass and metabolic rate during breeding. Surprisingly, the increased metabolic rate of high investment females did not result in higher levels of oxidative damage.

Both increased body mass and increased metabolic rate are likely to increase predation risk, through increasing food requirement whilst reducing escape ability. This study thus provides the first experimental evidence for metabolic costs of increased per offspring resource allocation, which are likely to be a key driver in the maintenance of variation in maternal reproductive investment.
